# Evaluation of a community-based participatory physical activity promotion project: effect on cardiovascular disease risk profiles of school employees

**DOI:** 10.1186/1471-2458-10-313

**Published:** 2010-06-05

**Authors:** Noha H Farag, William E Moore, David M Thompson, Cee E Kobza, Kathryn Abbott, June E Eichner

**Affiliations:** 1 University of Oklahoma Prevention Research Center, Oklahoma City, OK, USA; 2 Department of Biostatistics and Epidemiology, University of Oklahoma Health Sciences Center, Oklahoma City, OK, USA; 3 School Nurse, Anadarko Middle School, Anadarko, OK, USA; 4 School Nurse, East Elementary, Anadarko OK, USA

## Abstract

**Background:**

The efficacy of physical activity in improving cardiovascular disease (CVD) risk profiles has been well established. However, the effectiveness of health promotion programs implemented at the community level remains controversial. This study evaluated a school-based work-site physical activity program.

**Methods:**

Using a community-based participatory research model, a work-site wellness intervention was implemented in a rural public school system in Southwestern Oklahoma. During the 2005-2006 school year, 187 participants (mean age 45 years) completed a pre intervention screening for CVD risk factors followed by a physical activity promotion program. Post intervention screening was conducted after a 6 month period. During both screening sessions, body composition, blood pressure, lipids, glucose and self-reported physical activity levels were assessed. The focus of the intervention was on promoting physical activity. Opportunities for in school physical activity were created by marking hallways, adding a treadmill in each school, and allowing teachers to use planning periods for physical activity.

**Results:**

During the post intervention screening, compared to pre intervention levels, participants had lower total, low, and high density lipoprotein-cholesterol (t = 5.9, p < 0.0001, t = 2.6, p = 0.01, and t = 13.2, p < 0.0001 respectively), lower systolic blood pressure (t = 2.9, p = 0.004), and higher self-reported physical activity levels (Sign t = -1.901, p = 0.06).

**Conclusions:**

A successful participatory program was associated with improvements in several CVD risk factors among school employees. Limitations of this study such as seasonal variation in the outcome variables and lack of a control group limit our ability to draw solid conclusions about the effectiveness of the intervention.

## Background

Cardiovascular disease (CVD) is the leading cause of morbidity and mortality in the developed world [1]. Accordingly, the Healthy People 2010 Leading Health Indicators included factors known to play a major role in CVD risk [2]. Specifically, the top 2 indicators are physical activity and overweight/obesity, both of which play a major role in the initiation and progression of CVD. Currently, various funding agencies exert numerous efforts targeting these goals. Among such efforts are those funded by the Centers for Disease Control and Prevention (CDC) through their 33 Prevention Research Centers (PRCs) based in major universities throughout the U.S. [3]

The mission of the University of Oklahoma PRC is health promotion and disease prevention through the adoption of healthy lifestyles. To achieve this goal, a rural public school system was engaged in a community-based participatory research (CBPR) model. The processes involved in CBPR are described in detail elsewhere [4]. In short, actively involving the community in all stages of the research project and providing direct benefit to the community being studied makes CBPR an attractive research model for health promotion. The University of Oklahoma PRC focuses on increasing physical activity as a means of CVD prevention.

Numerous research investigations converge to provide evidence for the efficacy of physical activity in the reduction of CVD risk factors [5-7]. The Diabetes Prevention Program documented a 58% reduction in the incidence of diabetes following an intensive lifestyle intervention [7]. We are no longer concerned with proving, yet once again, the health benefits of physical activity but rather hope to provide useful models for implementation of intervention programs at the community level. However, without a detailed description of the processes involved in such interventions, it is difficult to build on one another's experiences.

Accordingly, this study evaluated a work-site wellness program which was implemented in a rural public school system in Oklahoma. Evaluation of the intervention focused on participation and retention rates, as well as improvements in CVD risk factors. In addition, a detailed description of the processes involved in the initiation and implementation of the wellness program as a collaborative effort between the PRC investigators and the public school district is provided.

## Methods

### Procedures

During a work-site screening conducted in spring 2004, we found that approximately 36% of participants had 4 or more CVD risk factors, and only 15% had no risk factors for CVD [8]. In response to concerns raised by the school nurses and the superintendent regarding the high prevalence of CVD risk factors, implementation of a work-site wellness program with follow-up to determine effectiveness of the intervention was considered. The program involved a bi-annual screening for CVD risk factors. Participants were given individual feedback regarding their results along with suggestions for improvement. A focus was placed on promoting an increase in physical activity levels, in particular physical activity levels that would meet or exceed the Surgeon General recommendations were promoted [9]. A post intervention screening was performed to assess the effectiveness of the intervention. All variables measured in the pre intervention screening were repeated during the post intervention screening. This was a community-based participatory research project that was initiated, designed, and implemented by the community with technical assistance from University investigators. Participants signed a consent form for the school acknowledging that the University of Oklahoma will have access to de-identified data for analysis and publication. The study was approved by the University of Oklahoma Health Sciences Center Human Research Protection Program as analysis of secondary data with an exemption from informed consent.

### Participants

The work-site wellness program was offered to employees of all five schools in the public school system in a rural school district in Southwestern Oklahoma. Of the 303 employees in all 5 schools, 202 participated in the baseline (pre intervention) screening. Demographic characteristics of the participants are displayed in Table 1. The school system operates in a town of 6,645 residents. According to the year 2000 census, the median annual household income was $ 36,570 with a 27% poverty rate. Low socioeconomic status and limited coverage and access to healthcare were cited by school nurses as possible reasons for the high prevalence of CVD risk factors among employees.

**Table 1:** Demographic characteristics and pre intervention levels of risk factors by sex

Mean ± SEM	Women N = 159	Men N = 43	P*
**Age **(years)	45 ± .81	45 ± 1.8	0.9

**Race **(% W/AI/AA)	77/17/4	79/16/5	0.9

**Body mass index **(kg/m^2^)	29.2 ± .61	29.3 ± .72	0.9

**Waist circumference **(cm)	91.8 ± 1.4	99.8 ± 2.3	0.005

**Body fat **(%)	37.9 ± .71	27.0 ± 1.0	0.0001

**Systolic BP **(mm Hg)	122 ± 1.3	130 ± 2.4	0.007

**Diastolic BP **(mm Hg)	75 ± .9	80 ± 1.7	0.009

**Heart rate **(beats/min)	74 ± .9	73 ± 1.9	0.6

**Triglycerides **(mg/dL)	128 ± 6.9	144 ± 11.7	0.3

**Total cholesterol **(mg/dL)	189 ± 2.7	207 ± 6.4	0.003

**HDL-cholesterol **(mg/dL)	57 ± 1.1	41 ± 1.6	0.0001

**LDL-cholesterol **(mg/dL)	107 ± 2.4	139 ± 5.9	0.0001

**Glucose **(mg/dL)	91 ± 1.8	91 ± 1.6	0.9

Race: W = white, AI = American Indian, AA = African American

* Test of between sex differences

### Intervention

After consultation with the school nurses and superintendent, it was agreed that promotion of physical activity would be the major focus of the intervention. An increase in physical activity levels that met or exceeded the Surgeon General's recommendation for at least 30 minutes/day of moderate levels of physical activity on 5 or more days of the week was promoted. Treadmills were purchased and placed in all 5 schools. Appropriately sized rooms were designated for the treadmills and made available at any time during the day. The schools allowed time during free periods for exercise. The school nurses used a measuring wheel (Rolatape^®^, Watseka, IL) to measure hallways and placed marks and posters that described, for example, that "walking up and down this hallway 4 times equals 1/8 mile". Participants were given the Walking Handbook (Cooper Aerobic Institute, Dallas, Tx) which is a 44 page booklet that promotes walking as a form of physical activity to maintain and improve health. It describes the health benefits of walking, provides a list of excuses for not walking and a commitment to walking contract. It describes getting started on a walking program and different rates and ways of walking. In addition, it provides a Cardiovascular Fitness Rating that assesses the time and fitness level of the walker, target heart rate ranges by age, and logs for tracking resting heart rate, weight loss and walking [10]. The handbooks were given to the participants for their personal use; they were not required to return the completed logs. Participants also received pedometers to facilitate self-monitoring of physical activity levels. To meet the Surgeon General's recommendation, the goal was set at 4,000 steps per day, which is equivalent to a half hour walk at a pace of 15 min/mile. A number of participants reported taking antihypertensives. However, the school nurses noted that compliance with antihypertensives was an issue among the employees. As part of the intervention, the nurses promoted compliance with antihypertensive medications.

### Measurements

The school nurses announced the initiation of the work-site wellness program and encouraged school employees to participate. The school nurses played the leading role in all aspects of the program. They conducted group and one-on-one promotional sessions to encourage participation in the program. They distributed flyers describing the benefits of participation and announcing the dates and times during which screenings were available. They scheduled all willing participants and explained the requirement for an overnight fast. Screenings were conducted twice. The baseline (pre intervention) screening was conducted in November 2005 and the post intervention screening was conducted in April 2006 of the same school year. The baseline (pre intervention) and post intervention screenings involved assessment of CVD risk factors including anthropometrics, blood pressure (BP), heart rate, lipid profile and fasting glucose:

#### Anthropometric assessments

Subjects stood barefoot during all anthropometric assessments. Body weight was measured to the nearest 0.1 kg on a digital scale. Height was measured to the nearest 0.1 centimeter using a portable stadiometer (Perspective Enterprises, Portage, MI). Body mass index (BMI) was calculated as weight in kg divided by the square of height in meters. Participant BMI was classified as follows; 18.5 kg/m^2 ^≤ Normal Weight < 25 kg/m2; 25 kg/m^2 ^≤ Overweight < 30 kg/m^2^; 30 kg/m^2 ^≤ Obese [11]. Waist circumference was measured using a tape measure at the midpoint between the upper iliac crest and lower costal margin in the midaxillary line. Body composition was determined by bioelectrical impedance using the Tanita TBF 300 (Tanita Corporation of America, Arlington Heights, IL).

#### Blood pressure and lipid assessments

BP and heart rate were measured using an automated device (HEM-907, Omron Healthcare Inc., Vernon Hills, IL). Two measurements were taken, using an appropriate size cuff, 1-min apart after a 3-min seated rest. After an overnight fast, capillary whole blood specimens were obtained by the finger stick method using heparin coated capillary tubes. The samples were analyzed using the Cholestech LDX system (Cholestech, Hayward, CA) immediately following sample collection. Samples were analyzed for total cholesterol, HDL-cholesterol, LDL-cholesterol, triglycerides, and glucose levels.

Individuals received results upon completion of their screening. Risk profiles were discussed individually and participants were given flyers describing the different measures taken, their significance in relation to CVD risk and targets for reducing their risk.

#### Questionnaires

Participants completed the short version of the International Physical Activity Questionnaire (IPAQ) [12] at pre- and post intervention. At pre intervention, they completed the IPAQ during the in-school screening session. At post intervention, they were given the questionnaire to complete and return to the school nurses. This was done because of time constraints of the teachers during the post screening sessions. The IPAQ is a self-administered questionnaire that assesses the level of health enhancing physical activity during a 7 day period. It is composed of 7 questions about frequency and duration of walking as well as daily activities that require physical efforts of moderate to vigorous intensities. Levels of energy expenditure were calculated as MET minutes/week (MET min/wk). METs are used to estimate the amount of oxygen used by the body during physical activity which gives an idea about the intensity of physical activity.

At the post intervention screening, participants completed a program evaluation questionnaire by answering yes or no to the following: "Has the adult wellness program changed your behavior in any positive way related to a conscious change or improvement in the following areas?: 1) Diet/Nutrition, 2) Exercise". Another question asked if they would be interested in participating in a similar program in the coming school year.

##### Program Implementation Costs

This project was a collaborative effort between the school nurses and University investigators. The school nurses were allowed to make employee wellness part of their jobs, but this added a load to their usual routine, especially on screening days. There were 5 schools and one make up day for those who missed the screening when it was provided in their school. Larger schools had 2 screening days. There were 8 days of screening per screening occasion (pre vs. post intervention). Five university personnel traveled on the 8 screening days for a 140 mile round trip at the University's mileage rate. The break down of the costs of the screening and intervention are outlined in Table 2.

**Table 2:** Cost of the Intervention.

Item	Total Price
Costs of Pre intervention Screening	

University Employee Salaries*	$16,468

School Nurse Salaries	$4,145

Travel	$2,716

Cholestech Analyzer × 2	$4000

Omron BP Monitor × 2	$1,200

Stadiometer	$400

Body Composition Analyzer	$1500

Scale	$400

Expendables @ $20/participant	$4,040

Total	$34,869

Costs of Intervention	

Treadmills @ $4900/site	$29,400

Pedometers @ $7/participant	$1,414
Walking booklets @ $12/participant	$2,424

Total	$33,238

* Salaries include fringe benefits and University's indirect cost rate charged on personnel salaries.

The cost of the post intervention screening is calculated by excluding the cost of equipment since they are reusable. The cost of post intervention screening was $27,369. The total cost of the intervention was approximately $95,000.

### Statistical Analysis

Data were analyzed using SPSS (SPSS for Windows, rel. 12.0, SPSS Chicago, IL). Sex differences in demographic and pre intervention risk factors were analyzed using t-test and chi-square statistics as appropriate. Paired t-tests were used to examine the differences between pre intervention and post intervention levels of risk factors and self-reported physical activity. To determine the possible effects of sex and antihypertensive use, they were included as between subjects factors in a series of repeated measures ANOVAs where the intervention was the within subjects factor. Self-reported physical activity data was highly skewed and was therefore analyzed using nonparametric tests. The sign test was used to examine the paired differences in physical activity levels pre-post intervention. Spearman's correlation was used to examine the relationship between pre-post intervention change in risk factor variables and pre-post intervention change in self-reported physical activity levels in MET min/wk. All tests were two-tailed and an a ≤ 0.05 was set for significance level.

## Results

### Descriptive characteristics of program participants

Demographic characteristics and pre and post intervention levels of risk factor variables are presented in Table 1. Of the 202 participants assessed during the pre intervention screening, 33% were classified as overweight and 37% as obese. The percentages of participants by risk category of each risk factor are displayed in Table 3.

**Table 3:** Percentage of program participants in risk categories at pre and post intervention screenings.

Risk Factor (%)	Pre intervention	Post intervention
**Body mass index **(kg/m^2^)		

Normal	30	32

Overweight	33	32

Obese	37	36

**Blood Pressure **(mmHg)		

Normotensive	42	43

Prehypertensive	47	51

Hypertensive	11	6

**Total Cholesterol **(mg/dL)		

Optimal	60	72

Borderline	30	25

High	10	3

**Glucose **(mg/dL)		

Normal	85	89

Prediabetic	12	8

Diabetic	3	3

### Pre-post intervention differences in risk factors

Of the 202 participants who completed the pre intervention screening, 187 also completed the post intervention screening (a 95% response rate). There were significant pre-post intervention differences in total cholesterol, LDL-cholesterol, HDL-cholesterol, and systolic BP (Table 4). During the post intervention screening, compared to pre intervention levels, participants had lower total cholesterol, LDL-cholesterol, HDL-cholesterol and systolic BP. A two-factor ANOVA (where time was a repeated factor and sex was a between subjects factor) yielded evidence of an interaction between sex and time for total cholesterol (F = 3.8, p = 0.05), LDL-cholesterol (F = 4.9, p = 0.03), and waist circumference (F = 4.0; p = 0.05). Men had a greater drop in total cholesterol and LDL-cholesterol compared to women (Table 4). In addition, women had a 2 cm decrease in waist circumference whereas men had a 1 cm increase in waist circumference on post intervention compared to pre intervention levels. There were no time by sex interactions for any of the other risk factors.

**Table 4:** Pre and post intervention differences in risk factors.

Mean ± SEM	Pre intervention	Post intervention	P-value	Cohen's d
Total Cholesterol (mg/dL)				
All	193 ± 2.6	181 ± 2.3	< 0.0001	0.4
Women	189 ± 2.7	178 ± 2.5	< 0.0001	0.4
Men	213 ± 6.7	191 ± 5.1	< 0.0001	0.6

HDL-Cholesterol (mg/dL)				
All	54 ± 1.1	47 ± 1.0	< 0.0001	0.5
Women	57 ± 1.2	49 ± 1.0	< 0.0001	0.6
Men	42 ± 1.5	35 ± 1.4	< 0.0001	0.8

LDL-Cholesterol (mg/dL)				
All	114 ± 2.5	109 ± 2.1	0.01	0.2
Women	107 ± 2.4	104 ± 2.3	0.1	0.1
Men	143 ± 6.3	130 ± 4.5	0.02	0.4

Triglyceride (mg/dL)				
All	130 ± 6.3	132 ± 5.4	0.8	0.03
Women	127 ± 7.1	128 ± 5.6	0.9	0.01
Men	145 ± 13.0	149 ± 15.1	0.7	0.1

Glucose (mg/dL)				
All	91 ± 1.6	91 ± 1.2	0.8	0.0
Women	91 ± 1.9	91 ± 1.4	1.0	0.0
Men	90 ± 1.7	92 ± 2.5	0.5	0.2

Systolic BP (mmHg)				
All	124 ± 1.2	121 ± 1.1	0.004	0.2
Women	122 ± 1.4	119 ± 1.3	0.001	0.2
Men	129 ± 2.7	130 ± 1.6	0.02	0.07

Diastolic BP (mmHg)				
All	76 ± 0.81	76 ± 0.75	0.7	0.0
Women	75 ± 0.88	75 ± 0.82	0.6	0.0
Men	80 ± 1.9	80 ± 1.7	0.7	0.0

Body mass index (kg/m^2^)				
All	29 ± 0.53	29 ± 0.55	0.3	0.0
Women	29 ± 0.63	29 ± 0.66	0.4	0.0
Men	29 ± 0.75	29 ± 0.76	0.1	

Waist circumference (cm)				
All	93 ± 1.3	93 ± 1.3	0.2	0.0
Women	92 ± 1.4	90 ± 1.4	0.01	0.0
Men	100 ± 2.6	101 ± 2.1	0.4	0.0

Fifty-one participants reported taking antihypertensive medications. To differentiate the effect of increased physical activity from improved compliance with antihypertensive medications, which was encouraged by the nurses, antihypertensive use was included as a between subjects factor in our repeated measures ANOVAs. There were no significant time × antihypertensive use interactions for any of the risk factors (all p's > 0.2). Furthermore, the data were analyzed excluding individuals on antihypertensive medications. The pre-post intervention differences remained significant despite the smaller sample size. Compared to pre intervention levels, post intervention levels of total cholesterol (t = 5.5, p < 0.0001), LDL-cholesterol (t = 2.9, p = 0.005), HDL-cholesterol (t = 10.5, p < 0.0001), and systolic blood pressure (t = 2.2, p = 0.03) remained significantly lower after excluding individuals taking antihypertensive medications.

### Risk factor changes in relation to self-reported change in physical activity

Only 126 participants returned the completed IPAQ. There was a statistically non-significant change in MET min/wk following the intervention (Sign Z = -1.901, p = 0.06; 2337, 95% CI: 1521-3152 vs. 2566, 95% CI: 1822 - 3309 MET min/wk at pre and post intervention respectively). However, there was no significant correlation between the change in self-reported physical activity levels and change in CVD risk factors. The highest correlation was that of SBP change and self- reported MET min/wk change (Spearman's ρ = 0.2, p = 0.04). To test the effect of the low response rate to the IPAQ questionnaire on our data, we examined the differences in outcome variables (pre-post change) between responders and non-responders to the IPAQ. There were no significant differences between responders and non-responders in any of the CVD risk factor changes (Table 5). There were also no differences in the pre intervention levels of physical activity between responders and non responders to the IPAQ (MET min/wk; 2327 ± 415 and 2790 ± 623 in responders and non responders respectively, Z = -0.07, p = 0.9).

**Table 5:** Differences between responders and non responders to the IPAQ questionnaires in the amount of change in major cardiovascular disease risk factors following the intervention.

Mean ± SEM	Responders	Non Responders	t-statistic	P- value
	N = 126	N = 61		
**Body Mass Index (kg/m^2^)**	0.25 ± 0.2	0.21 ± 0.7	0.1	0.9

**Total Cholesterol Change (mg/dL)**	- 11.7 ± 2.5	- 15.1 ± 4.1	-7.4	0.5

**LDL-Cholesterol Change (mg/dL)**	-4.3 ± 2.2	-6.4 ± 3.4	- 0.5	0.6

**HDL Cholesterol Change (mg/dL)**	-7.1 ± 0.7	- 8.8 ± 1.1	-1.5	0.1

**Systolic BP Change (mmHg)**	- 3.2 ± 1.1	- 1.5 ± 1.4	0.9	0.4

#### Participant evaluation of the intervention

One-hundred and thirteen out of 187 participants returned the program evaluation questionnaire. A positive change in diet was reported by 40% of participants while 35% of participants reported a positive change in exercise. Eighty five percent of participants indicated an interest in participating in a similar program the next time it is offered.

## Discussion

Following the intervention there was a significant decrease in total cholesterol, LDL-cholesterol, HDL-cholesterol, and systolic BP compared to pre intervention levels. The sample was comprised mostly of women making a sex comparison difficult, but because of the known sex differences in lipid levels [13], the data was analyzed for possible pre-post intervention differences by sex. Men had significantly larger drops in total cholesterol and LDL-cholesterol compared to women.

The decrease in HDL-cholesterol following the intervention was unexpected. However, the replacement of saturated fats with carbohydrates or polyunsaturated fats has been found to be associated with reductions in HDL-cholesterol levels [14,15]. However, it can not be confirmed that this is the case in this study due to a lack of detailed dietary data from participants. In a meta-analysis of randomized-controlled trials, Kodama et al. documented that a minimum exercise length and energy expenditure levels are needed to produce positive effects on HDL-cholesterol levels [16]. They also demonstrated that exercise effects on HDL-cholesterol are more pronounced in subjects with low BMI. In the current study, only 17% of participants performed exercise that fell in the vigorous activity range (≥ 3000 MET m/wk), based on responses to the IPAQ. In addition, 70% of the participants in the current study were either overweight or obese. These issues together with seasonal variation in blood lipid levels, may explain this observed effect on HDL-cholesterol [17]. Future annual follow-ups should be conducted in the fall to alleviate concerns about seasonal variations in risk factors.

The increase in self-reported physical activity following the intervention was not statistically significant. However, the change in self-reported physical activity levels was not correlated with the change in CVD risk factors. Self-reported compliance is an issue in and of itself because people tend to over-report changes in exercise levels. Thus, the possibility can not be excluded that the moderately positive changes that were observed on CVD risk factors were due to the effect of the repeated measurement itself as opposed to an actual change in physical activity levels. It has been shown that repeated population screening is associated with reductions in CVD risk factors in high risk individuals [18]. A Hawthorne effect whereby the mere presence of the intervention, not the intervention itself, is associated with favorable changes in outcome measures can therefore not be excluded. This is because people tend to work harder to make lifestyle changes when they are participants in an experiment because of the attention they receive, not because of the experimental manipulation itself. In addition, BMI did not change following the intervention. However, favorable changes in CVD risk profiles have been documented in response to an increase in physical activity levels despite a lack of effect on body weight [19].

Certain limitations of this study are worth noting. The screenings were conducted during different seasons in the same school year. Due to the seasonal variation in blood lipids and blood pressure [17,20], these results should be taken with caution. To alleviate concern regarding seasonal variation, there is a plan of continuing the wellness program while conducting an annual screening in the fall of each school year. However, seasonal variation is related to differences in temperature, which were not a concern in this study since both pre and post assessments were conducted in the spring and late fall when extreme temperatures were not an issue.

One strength of this study is that two-third of the employees of the public school system chose to participate in the wellness program. However, we do not have information on those who chose to not take part in the screenings or walking program. The external validity of findings from research studies rests on the assumption that the participants represent the populations from which they were drawn. However, biases associated with non-response and drop-out rates may often result in overestimation of the effect of the interventions. It is quite possible that the changes we observed were caused by a "healthy participant effect" whereby participants report healthier lifestyles than non participants and are thus more likely to comply with the study protocol. This issue could be addressed if we had information on the fraction of the public school system employees who chose not to participate. However, this information is not available and their choice not to participate prohibited us from accessing any information related to them such as socioeconomic status, race, age or any other demographic information from which we could draw conclusion about the characteristics of the non-responders.

A high non-response rate to the IPAQ questionnaire at post intervention was most likely caused by allowing participants to complete it at a later time and not during the actual in-school screening. Although completing it during the screening sessions would have increased compliance, as it did with the pre intervention IPAQ questionnaires, this was not possible because the nurses wanted the screening sessions to be as short as possible because of time constraints of the teachers towards the end of the school year. Nevertheless, this high non-response rate raises the suspicion of a nonresponse bias. However, there were no significant pre-post intervention differences between responders and nonresponders in any of the CVD risk factors of interest. In addition, we did not have enough men in the sample to permit an adequate sex comparison. However, the composition of the work force in that public school system is such that there are a larger number of women than men employees. Finally, objective data on physical activity were not collected. The fact that self-report of exercise creates a smaller burden on the participants made the IPAQ a more suitable assessment method for this study. In addition, self-reported physical activity predicts cardiorespiratory fitness [21,22]. Finally, lack of a control group limits the validity of the current findings. However, because of the participatory nature of this work and the desire of the school community to improve the health of as many interested employees as possible, it was not possible to randomize participants to a non-intervention arm. In addition, limited funds hindered our ability to involve a non-intervention group in another community.

The main strength of this community-based participatory research project was in the ability to interest two thirds of the public school employees in a physical activity promotion program, while maintaining a 95% response at 6-months follow-up. It is well recognized that physical activity is effective in reducing CVD risk. However, getting individuals to increase their activity levels is a challenging task that, along with changes in dietary factors, has sustained the obesity epidemic in the U.S. Community-based programs are needed to increase awareness and compliance with physical activity recommendations. If we exclude salaries of University investigators from the project costs, approximately $60,000 would be needed to implement this study at the community level. However, consideration needs to be given to the fact that the work of the 5 investigators would need to be conducted by the school nurses which means that they would need 29 full days to screen the 202 participants. This is not feasible, since the 2 nurses are serving 5 schools. If implementation of health promotion activities is to take place in the "real-world" using available resources, serious consideration needs to be given to policies affecting the availability of personnel and funds to support these efforts.

## Conclusion

Despite the above limitations, this study demonstrated the effectiveness of a partnership between researchers and public schools in an effort to improve the health of school employees. Moderate effects on CVD risk profiles following a physical activity program instituted by community members and evaluated by academic researchers were documented. The 6-month wellness program instituted by public school nurses was associated with a modest increase in self-reported physical activity levels, a drop in total cholesterol, LDL- cholesterol and systolic BP. However, the results should be interpreted with caution given the above mentioned limitations of this study. Longer follow-up is needed to determine if these effects were sustained over time. The CBPR process may be an effective health promotion delivery strategy for improved cardiovascular health in underserved communities.

## Competing interests

The authors declare that they have no competing interests.

## Authors' contributions

NF participated in the conception and design of the study, assisted with data collection analyzed the data and wrote the manuscript. WM assisted the with data collection, evaluated the statistical analyses of the data and participated in drafting the manuscript. DT evaluated the statistical analyses and participated in drafting the manuscript. CK participated in the conception and design of the study, the data collection and evaluated the written manuscript. KA participated in the conception and design of the study, the data collection and evaluated the written manuscript. JE participated in the conception and design of the study, assisted in data collection, drafting the manuscript and gave final approval for the version to be published. All authors have read and approved the final version of the manuscript.

## Pre-publication history

The pre-publication history for this paper can be accessed here:

http://www.biomedcentral.com/1471-2458/10/313/prepub
